# Symptomatic renal arteriovenous malformation before and after rupture **--** endovascular and surgical approach

**DOI:** 10.1016/j.eucr.2025.103106

**Published:** 2025-06-16

**Authors:** Lisa Meunier-Geleng, Loic Choffel, Gabriel Simon, Alexandre Frontczak, François Kleinclauss

**Affiliations:** a Urology Department, University Hospital Center of Besançon, 3, Boulevard Fleming, Besançon, 25000, France; b University of Franche-Comte, Besançon, France; c Department of Radiology, University Hospital of Besançon, 25030 Besançon, France

**Keywords:** Renal arteriovenous malformations, Rupture, Endovascular embolization, Nephrectomy

## Abstract

Renal arteriovenous malformations are rare, with significant challenges in management due to their high-flow nature and potential serious complications. We present a case of a 78-year-old female with no significant medical history, who presented with hematuria and right-sided lumbar pain. Imaging revealed a large right renal arteriovenous malformation. Due the size and the location, a surgical approach by right nephrectomy rather than endovascular therapy was decided. Prior to surgery, the renal arteriovenous malformation ruptured into the renal excretory cavities, with a retroperitoneal aneurysm causing a large hematoma and hematuria. Emergency embolization was performed, followed by a nephrectomy the next day.

## 1 Introduction

Renal arteriovenous malformations (AVMs) are rare vascular anomalies in which arteries and veins connect abnormally, disrupting normal blood flow to the kidneys and potentially leading to various complications. While most renal AVMs are acquired due to renal trauma,^1^ they can also be congenital^2^ or idiopathic. These malformations are often asymptomatic, though they may cause symptoms such as hematuria^3^^,^^4^, hypertension^5^, and flank pain in some cases. Most case reports focus on small-caliber renal arteriovenous malformations, for which endovascular treatment using coils or Amplatzer vascular plugs is typically recommended.^6^ Management may differ when a nidus is present, which is the central core of an AVM where the anomalous veins and arteries converge. In this case, additional occlusion techniques, such as gelfoam, alcohol, or viscous liquid embolics, are employed.^2^

There are few documented cases of large renal arteriovenous malformations. While one might assume they require surgical intervention, some cases have shown that embolization alone can be both sufficient and safe.^7^ Surgery remains a rare option but may be necessary in cases of ruptured renal arteriovenous malformations ^8^ when embolization is unsuccessful ^9^ or in large malformations where there is an increased risk of embolic material migration, as seen in our case.

## 2 Case presentation

A 78-year-old female patient presented to the emergency department with hematuria associated with right-sided lumbar pain since the previous day. She had no significant past medical history except for medically treated arterial hypertension. There was no history of renal trauma, renal biopsy or surgery, nor family history of hereditary or bleeding disorders.

**Fig. 1:**
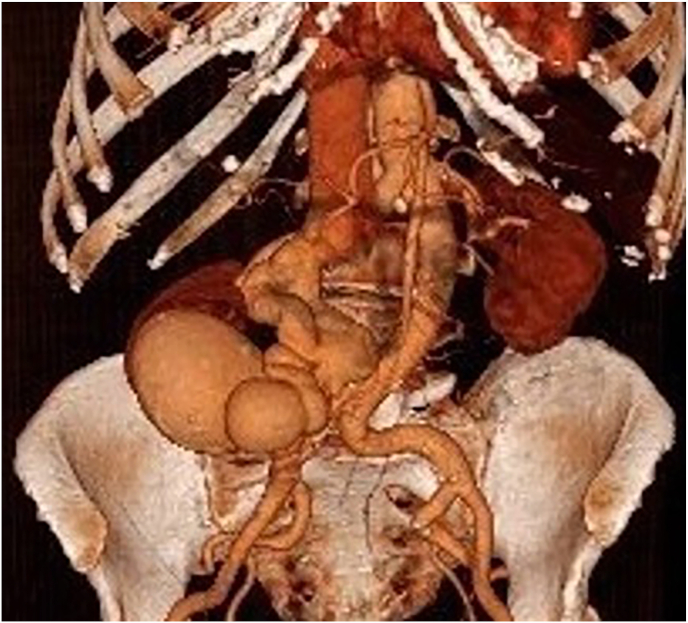
Pre rupture 3D image of the renal AVM A 3D volume-rendered image from the CT data showing the nidus of the renal arteriovenous malformation. The image demonstrates the right renal artery and vein entering the nidus, with noticeable dilation caused by the increased blood flow volume.

On admission, the patient was hemodynamically stable. The initial physical examination revealed percussion pain in the right renal region associated with hematuria and a palpable thrill in the right lumbar region. No other systemic abnormalities were noted. Laboratory tests such as blood and biochemical analysis showed hemoglobin of 11.5 g/dL and creatinine level of 102 μmol/L, there were no signs of inflammation and coagulation parameters were within the normal range. A computed tomography scan (CT scan) was performed and showed a large right renal arteriovenous malformation including a nidus measuring 8 × 8 cm with a renal arteria measuring 15 mm and a renal vein measuring 22 mm (Fig. 1).

Initial management consisted of bladder catheterization with continuous irrigation and blood pressure control with a systolic target inferior to 140 mmHg. Continuous monitoring in intensive care was carried out, as well as a pre-operative anaesthetic assessment with an echocardiography in the event of an aortic clamping during the surgical procedure.

On account of the large size and high flow of the malformation, an endovascular therapy seemed unfavourable due the possibility of incomplete occlusion and migration of embolizing material. A surgical approach by total right nephrectomy was preferred.

On the eve of surgery, the patient experienced an abrupt arterial hypertension to 180/70 mmHg followed by hypotension and abdominal pain, without tachycardia or desaturation. The blood count showed a sudden decrease of hemoglobin to 6.5 g/dl requiring blood transfusion.

**Fig. 2:**
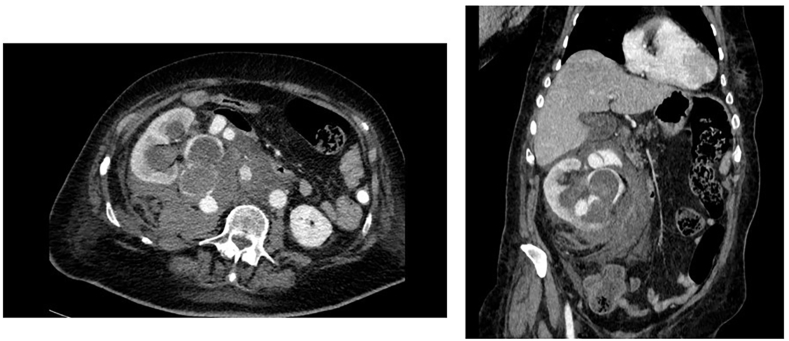
Axial and coronal post rupture CT scan image CT scan image showing retroperitoneal rupture of the right renal arteriovenous malformation and of the excretory tract responsible of a voluminous retroperitoneal hematoma.

A CT scan was performed and revealed a rupture of the right renal arteriovenous malformation within the right excretory cavities with formation of a retroperitoneal aneurysm measuring 25 mm in diameter. This resulted in a voluminous retroperitoneal hematoma with mass effect on adjacent structures (Fig. 2).

**Fig. 3:**
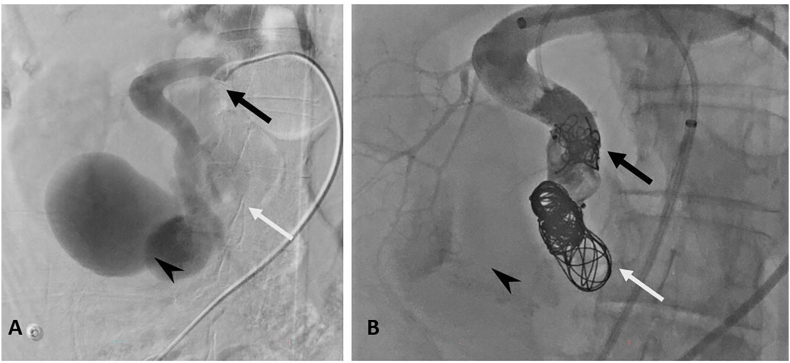
Right renal angiography before and after embolization **A.** Coronal angiography with catheter (black arrow) positioned in the renal artery, showing the nidus of the malformation (arrowhead) and an efferent vein (white arrow). Note the highly circulating nature of the malformation, with rarefaction of branches destined for the rest of the upper polar renal parenchyma. **B.** Angiography in the coronal plane after arterial embolization with plug, coil and glue (black arrow), venous embolization with coil (white arrow), showing no circulation in the malformation (arrowhead). Note the redistribution of flow in the branches to the rest of the upper polar renal parenchyma.

The patient underwent an emergency embolization. Using the right common femoral artery approach, artery occlusion was performed using packing coils and a 22 × 18mm Amplatzer Vascular Plug II. Then complementary renal vein occlusion was performed after retrograde puncture of the right common femoral vein by using packing coils. Additional occlusion was made through the arterial plug using a 1:2 Glubran-lipiodol mixture until the plug was filled. Occlusion of the entrance and exit of the renal AVM was essential in order to prevent retrograde migration of the embolic agent into the vein. A control serigraphy confirmed the exclusion of the high flow arteriovenous fistula (Fig. 3).

**Despite successful haemodynamic stabilisation following embolization, the patient remained dependent on vasopressors and required blood transfusions. Moreover, she presented persistent flank pain and gross clotting hematuria. Given these ongoing issues and the risk of further complications, a total right nephrectomy via median laparotomy was ultimately performed the following morning.** The procedure did not present any peri-operative complications, hem-o-loks clips were placed in the interaortocaval region without difficulty, as the embolization material was located distally and did not interfere with the nephrectomy. Control of the renal AVM was achieved by a lateral clamping of the vena cava with a vascular clamp.

**Fig. 4:**
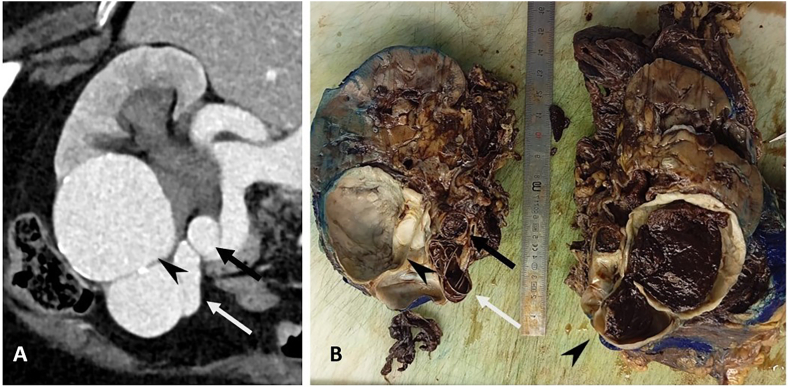
Pre-embolization CT scan and post-embolization excision specimen of the right kidney. **A.** Portal CT view after MPR reformatting of the right kidney, showing a feeder artery arising from the renal artery (black arrow), a bilobed nidus (arrowhead) and an efferent vein draining into the renal vein (white arrow). **B.** Nephrectomy block, post-embolization with section in the long axis of the kidney, showing the same feeder artery embolized by plug, coil and glue (black arrow) and an efferent vein embolized by coil (white arrow). Note the partially thrombosed nidus (arrowhead).

Anatomopathology confirmed an arteriovenous malformation of the renal hilum ruptured in the excretory urinary cavities and in the retroperitoneum. No signs of malignancy were observed (Fig. 4).

## 3 Discussion

Due to their rarity, large arteriovenous malformations are challenging to manage. Whilst the majority remain asymptomatic, they can sometimes present significant symptoms such as hematuria, high blood pressure or flank pain. In severe cases, heart failure can occur over time as the direct shunting of blood increases the overall volume returning to the heart causing left ventricular dilatation and increased preload.^10^ Large AVMs are at even greater risk of complications such as rupture ^8^ which is why they require prompt treatment to prevent life-threatening outcomes.

This case highlights how AVMs represent a severe condition while sometimes having fairly common symptoms. It allows to show the importance of considering renal AVMs in the differential diagnosis of unexplained hematuria and flank pain, even in the absence of known risk factors, such as a history of renal trauma^11^ or genetic disorders^12^.

The management of renal AVMs depends on the size, location, and symptoms associated with the malformation. The preferred option is endovascular therapy as it is less invasive and allows renal function to be preserved.^13^^,^^14^ However, in our case the decision to proceed with a surgical approach by nephrectomy was based on the size of the fistula and its localization in the renal hilum, which constituted a unique challenge.

The patient's sudden hypotension and drop in hemoglobin, along with the evidence of a ruptured AVM further complicated the clinical picture.

One thing that could have been handled differently is the introduction of continuous hypertensive treatment while awaiting surgery. Hypertensive treatment was only given when she had systolic hypertensive peaks superior to 140 mmHg. The patient reported being stressed the night before surgery and hypertensive peaks may have played a role in the rupture. Applying a strict blood pressure control protocol could have perhaps prevented the complication.

To go further, our surgical procedure was scheduled a few days after admission as the patient did not present severity criteria. The rupture of the AVM into the excretory cavities and the massive retroperitoneum hematoma underlined the necessity to treat as soon as the first symptoms appear.

Emergency embolization successfully occluded the high-flow fistula, reducing the risk of further haemorrhage and allowed to stabilise temporarily the patient. The embolization procedure, which involved the use of coils and Amplatzer plugs in the renal arteria as well as the renal vein to prevent material migration, is an effective approach for managing renal AVMs, particularly in cases where nephrectomy is not immediately necessary^15^, ^16^, ^17^.

However, given the size of the AVM and the patient's overall condition, a nephrectomy was ultimately required to prevent further complications. Surgery remains a treatment of choice in cases where embolization seems not sufficient or feasible.

## 4 Conclusion

Throughout this case, large renal AVM proved how challenging their management can be, especially as there are few cases to refer to in the literature.

Strict blood pressure control protocols and timely intervention, including emergency embolization and subsequent nephrectomy, proved to be essential in managing this complex case and preventing further complications. It highlights the need for a tailored, multidisciplinary approach to the management of renal AVMs, ensuring the best possible outcome for the patient.

## CRediT authorship contribution statement

**Lisa Meunier-Geleng:** Writing – review & editing, Writing – original draft, Resources, Project administration, Methodology, Investigation, Formal analysis, Conceptualization. **Loic Choffel:** Supervision. **Gabriel Simon:** Writing – original draft. **Alexandre Frontczak:** Validation. **François Kleinclauss:** Validation.

## Conflicts of interest

The authors have no competing interests.
